# Ultrasound-guided transperineal vs transrectal prostate biopsy: A meta-analysis of diagnostic accuracy and complication rates

**DOI:** 10.1515/med-2024-1039

**Published:** 2024-10-02

**Authors:** Tao Wu, Yanchun Xing

**Affiliations:** Department of Urology, Huangshan City People’s Hospital, Huangshan , 245000, China; Department of Medicine, Huangshan Vocational Technical College, Huangshan, 245000, China

**Keywords:** prostate cancer, ultrasonic guidance, prostatic needle biopsy, meta-analysis, systematic evaluation

## Abstract

**Objectives:**

We conducted a systematic review to compare the diagnostic utility of ultrasound-guided transperineal (TP) and transrectal (TR) prostate biopsy methods for prostate cancer detection.

**Methods:**

We searched PubMed, Embase, Web of Science, and Cochrane databases up to October 30, 2023, for relevant studies, screening the literature and assessing bias independently.

**Results:**

Eleven trials were analyzed using relative risk and 95% confidence intervals, with no evidence of publication bias. Diagnostic rates showed no significant difference between TP and TR biopsies (mean difference [MD]: 1.03, 95% confidence interval [CI]: 0.91–1.14, *P* = 0.56). Prostate volume analysis also showed no significant difference (MD: –0.07, 95% CI: –0.73 to 0.59, *P* < 0.0001, combined effect size *P* = 0.83). Similarly, PSA levels were comparable between TP and TR biopsies (MD: 0.93, 95% CI: –0.44 to 2.30, *P* < 0.0001, combined effect size *P* = 0.18).

**Conclusion:**

Both biopsy methods exhibit similar diagnostic accuracy; however, TP has a lower risk of biopsy

## 1 Introduction

Prostate cancer (PCa) represents one of the most prevalent malignancies afflicting the male genitourinary system. As reported by Van Poppel et al., projections of cancer mortality within the European Union indicate that PCa occupies the third position in terms of mortality rates among all malignant tumors, with an anticipated 766,200 male fatalities [1]. Howrey et al. have demonstrated that the incidence of PCa is the highest among male malignant tumors in the United States, with mortality rates second only to lung cancer [2]. In China, Fu et al. identified approximately 72,000 new cases of PCa in 2015, yielding a crude incidence rate of 10.23 per 100,000 individuals. The incidence rates of PCa in China and globally stand at 6.59 and 6.47 per 100,000, respectively, placing it sixth among male malignant tumors [3].

PCa is a globally prevalent malignancy; yet, the optimal methods for its detection and diagnosis remain subjects of debate. Prostate-specific antigen (PSA) screening is commonly employed, despite the traditional biopsy threshold’s association with a positive predictive value of only 20–30%, leading to numerous unnecessary biopsies [4,5]. Kayano et al. have identified transrectal ultrasound (TRUS)-guided prostate biopsy as a pivotal diagnostic tool for PCa [6]. Nonetheless, conventional prostate biopsy procedures are not without limitations, including the risk of serious complications and a considerable false-negative rate [7]. This underscores the critical need to establish the most efficacious and secure means of diagnosing PCa.

Despite its widespread recognition as the standard method for detecting PCa and its decades-long global use, systematic transrectal (TR) prostate biopsy has been found by Xue et al. to significantly underreport the initial incidence of PCa, with a rate of malignant detection as high as 49% [8]. Furthermore, this procedure is associated with potentially serious complications, such as rectal bleeding, fever, hematuria, and acute urinary retention [9,10,11]. Recognizing the heightened risk of false negatives and complications with TR biopsy, Ding et al. have proposed the use of ultrasound-guided transperineal (TP) examination as a strategy to enhance both the detection rate and safety of prostate biopsy [12].

Despite the proliferation of studies that compare the detection rates and reliability of ultrasound-guided TP and TR prostate biopsy methods, controversy persists regarding the efficacy of both techniques. Consequently, a meta-analysis is warranted to furnish a higher level of evidence and to elucidate definitive conclusions regarding the two biopsy modalities. Prior meta-analyses have aggregated observational studies and randomized controlled trials (RCTs) without accounting for the nuances in study design and quality assessment methods, thereby introducing heterogeneity in the results. To achieve more precise and uniform conclusions, we have conducted a meticulous evaluation of study quality and have separately pooled observational and randomized controlled studies. Moreover, we have systematically reviewed all relevant studies to appraise the consistency of the two biopsy methods.

## 2 Methods

### 2.1 Inclusion criteria

The study design is an RCT or cohort/case–control study. The study subjects are patients undergoing prostate biopsy. The methods of intervention are ultrasound-guided perineal puncture and TR approach. Additional considerations are as follows: the number of biopsy samples and the catheterization method were maintained constant across all intervention groups. Outcomes are PCa diagnosis rate, prostate volume, and PSA level.

### 2.2 Exclusion criteria

The literature has no available information or incomplete data. The literature is not original research. Patients with a prior history of PCa, acute prostatitis, or a proven urinary tract infection are excluded.

### 2.3 Literature search

We conducted a comprehensive search of the PubMed, Embase, Web of Science, and Cochrane databases to identify RCTs, cohort studies, or case–control studies that assessed the diagnostic efficacy of ultrasound-guided TP and TR prostate biopsy in the detection of PCa. Our literature review was conducted up to October 30, 2023, adhering to stringent inclusion and exclusion criteria to ensure the selection of relevant studies.

To enhance the recall and precision of our search, we employed a fuzzy search strategy that included the references of the included studies, aiming to capture all pertinent RCTs, cohort studies, or case–control studies that met our inclusion criteria. Our search strategy was formulated as follows: Search ((((((transperineal) OR (transrectal)) AND (prostate biopsy)) AND (detection)) OR (detection)) AND (prostate cancer)) OR (prostatic neoplasms).

### 2.4 Literature screening and data extraction

The authors compressed the data for the included studies and used pre-determined statistical tables. Any differences are settled by discussion. Important aspects of the study included: the first author’s last name, year of publication, patient age, study design, study population, number of patients in both groups, patients’ PSA levels and prostate volume, biopsy method, and covariates analyzed.

### 2.5 Quality evaluation

The authors evaluated the quality of observational studies using the Newcastle–Ottawa Scale (NOS). Studies scoring below 7 were deemed of low quality and were consequently excluded from the analysis. Among the RCTs, only one study by Udeh et al. was excluded due to a high loss bias, as a quarter of the patients lost follow-up [13]. Differences in authors’ opinions were resolved through consensus. In the absence of consensus, a third of the experts were invited to provide input to resolve the issue.

### 2.6 Statistical analysis

Our meta-analysis employed ReviewManager software (version 5.4) to process all statistical data. We evaluated the degree of difference between TR and TP methods within 95% confidence intervals (CIs) using a combined odds ratio (OR). The heterogeneity hypothesis was validated through the calculation of the chi-square test and the *I* ^2^ statistic.

In this meta-analysis, we utilized both the fixed effects model (Mantel–Haenszel method) and the random effects model (DerSimonian–Laird method) for analysis. If significant heterogeneity (*I* ^2^ > 50%) was detected, we employed the random effects model. In the absence of significant heterogeneity, the fixed effects model was utilized. Moreover, if substantial heterogeneity was identified between studies, we conducted an exploration of the potential sources of heterogeneity.

Sensitivity analyses were performed by sequentially excluding individual studies to assess the robustness of our findings. A *P*-value of <0.05 was considered indicative of statistically significant differences.

## 3 Results

### 3.1 Literature screening results and basic characteristics

**Figure 1:**
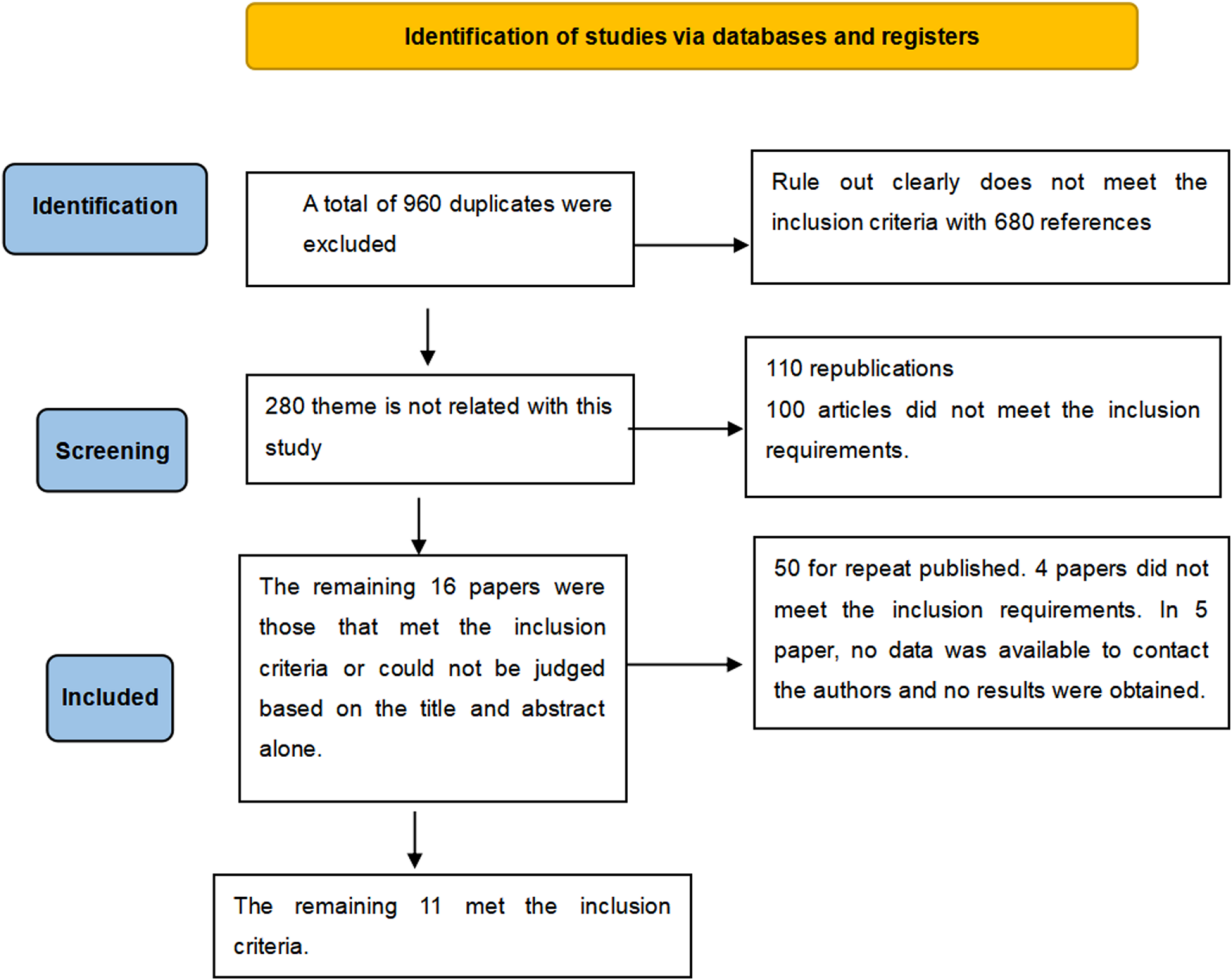
Literature screening process and results.

The research screening process is depicted in Figure 1. Initially, 960 duplicate literature entries were identified and excluded, and the remaining articles were selected based on their titles and abstracts. Two independent reviewers were chosen for this task. Subsequently, 680 articles were deemed unsuitable due to their clear non-compliance with the inclusion criteria, 210 were found to be duplicates, and 50 did not fulfill the inclusion requirements. This left 20 articles that either met the inclusion criteria or could not be assessed solely based on the title and abstract. Full-text versions of the remaining 20 articles were obtained, and the same two reviewers independently assessed whether each article should be included. Any discrepancies were resolved through discussion. After this process, 2 articles were excluded, 6 did not meet the inclusion criteria, and 1 could not be included due to the unavailability of data and the inability to contact the authors. Ultimately, 11 articles met the inclusion criteria and were included in this systematic review.

### 3.2 Comparison of included literature

Table 1 illustrates the variability in study populations and sample sizes across different studies. Among these, the studies by Lu et al. [15] and Di Franco et al. [18] featured relatively substantial populations and sample sizes, while the study by Pepe et al. [23] had a smaller sample size of only 200 cases. The PSA levels also exhibited variations between the TP and TR groups. For instance, Lu et al. [15] reported a PSA level of 22.0 ng/mL in the TP group and 23.2 ng/mL in the TR group. Similarly, Song et al. [16] found that the PSA level in the TP group was 7.49 ng/mL, compared to 7.18 ng/mL in the TR group. These discrepancies could be attributed to differences in disease distribution or biopsy strategies. Prostate volume also displayed variations between the TP and TR groups. Lu et al. [15] reported a prostate volume of 57.0 mL in the TP group and 54.4 mL in the TR group. Abdollah et al. [17] reported a prostate volume of 65.4 mL in the TP group and 62.3 mL in the TR group. These variations could be due to differences in disease distribution or biopsy strategies. Regarding biopsy methods, some studies employed different techniques. Abdollah et al. [17] used the ultrasound-guided saturation prostate rebiopsy technique. Di Franco et al. [18], Shida et al. [19], and Pepe et al. [23] utilized the mpMRI/TRUS fusion-targeted biopsy technique. These different biopsy techniques could have influenced the study results. In terms of covariates, most studies considered factors such as age, PSA, prostate volume, DRE, and the results of previous biopsies. These factors were used to adjust the findings to mitigate potential bias and the impact of confounding factors. Regarding NOS scores, the studies ranged between 7 and 9, indicating a relatively high quality of the studies. Table 2 presents a comparative study of ultrasound-guided TP and TR prostate biopsy. The table lists the year of each study, patient age, study population, sample size, PSA level, prostate volume, and biopsy method. Guo et al. [14] conducted a study in 2015 with sample sizes of 173 (TP) and 166 (TR), PSA levels of 8.81 and 10.48, respectively, and prostate volumes of 47.2 and 45.9, respectively. They employed a 12-core systematic biopsy method. The 2019 study by Jiang et al. involved a larger sample size of 2,962 men, with 1,216 undergoing TR biopsies and 1,746 undergoing TP biopsies. PSA levels and prostate volume also differed between the two groups. In the 2014 study by Cerruto et al., with a sample size of 54 (TP) and 54 (TR), PSA levels and prostate volume also varied between the two groups. They used a 14-core systematic biopsy method. The basic characteristics are shown in Tables 1 and 2.

**Table 1:** Comparisons of the observational characteristics of ultrasound-guided transperineal and transrectal prostate biopsy

Study	Year	Age	Study design	Study population	Patients		PSA level (ng/mL)		Prostate volume (mL)		Biopsy methods	Covariates	NOS score
					TP	TR	TP	TR	TP	TR			
Lu et al. [15]	2023	70.1	Cohort study	Compared TR or TP acceptance between June 2017 and September 2021	245	207	22.0	23.2	57.0	54.4	TP OR TR	Age, PSA, PV, DRE, histologic findings on previous biopsy	9
Song et al. [16]	2022	65.20	Cohort study	10,901 cases between May 2003 and December-December 2017	561	561	7.49	7.18	44.30	43.29	TP OR TR	Age, PSA, PV, DRE, histologic findings on previous biopsy	8
Abdollah et al. [17]	2010	63.3	Cohort study	Patients who underwent a rebiopsy between 2005.9 and 2008.6	140	140	9.7	10	65.4	62.3	Ultrasound-guided saturate prostate rebiopsy	Age, PSA, PV, DRE, histologic findings on previous biopsy, the number of previous negative biopsy sets	7
Di Franco CA et al. [18]	2017	68	Cohort study	219 men who underwent prostate biopsies between 2004 and 2014	111	108	6.9	7.8	55	43	TP OR TR	Age, PSA, PV, DRE, histologic findings on previous biopsy	8
Shida et al. [19]	2016	67	Cohort study	1,019 patients underwent the first prostate biopsy and 298 repeated prostate biopsies	66	113	6.7	9.1	37	46	TP OR TR	Age, PSA, PV, DRE, histologic findings on previous biopsy	9
Lo et al. [20]	2019	69	Cohort study	Retrospective study of 100 TPUSPBs and 100 TRUSPBs.	35	25	9.5	12.0	56.8	46.2	TP OR TR	Age, PSA, PV, DRE, histologic findings on previous biopsy	8
Miano et al. [21]	2014	64.6	Cohort study	From January 2015 to January 2016, there were 200 men	278	255	8.6	8.6	38.9	42.4	TP OR TR	Age, PSA, PV, DRE, histologic findings on previous biopsy	7
Pepe et al. [23]	2016	61	Cohort study	Patients persistently suspicious of PCa between 2015.1 and 2016.1	200		86	/	/	/	mpMRI/TRUS fusion-targeted biopsy	Self-control	8

Note: TR is ultrasound-guided perineal prostate biopsy; TP is ultrasound-guided transrectal prostate biopsy.

**Table 2:** Comparisons of randomized controlled trials of ultrasound-guided transperineal and transrectal prostate biopsy

Study	Year	Age	Study population	Patients		PSA level		Prostate volume		Biopsy methods
				TP	TR	TP	TR	TP	TR	
Guo et al. [14]	2015	67	Patients between 2012.6 and 2014.8 with a PSA >4.0 ng/mL	173	166	8.81	10.48	47.2	45.9	Systematic 12-core biopsy
Jiang et al. [22]	2019	68 in TP group, 71 in TR group	2,962 men who underwent transrectal (=1,216) or transperineal (=1,746) system 12-core prostate biopsy	1,216	1,746	40.31	38.02	59.64	51.64	TP OR TR
Cerruto et al. [24]	2014	66.5 in TP group, 67.3 in TR group	Consecutive patients with a PSA >4 ng/mL	54	54	15.95	12.36	56.29	61.49	Systematic 14-core initial prostatic biopsy

Note: TR is ultrasound-guided perineal prostate biopsy; TP is ultrasound-guided transrectal prostate biopsy.

### 3.3 Comparison of PCa diagnosis rate

**Figure 2:**
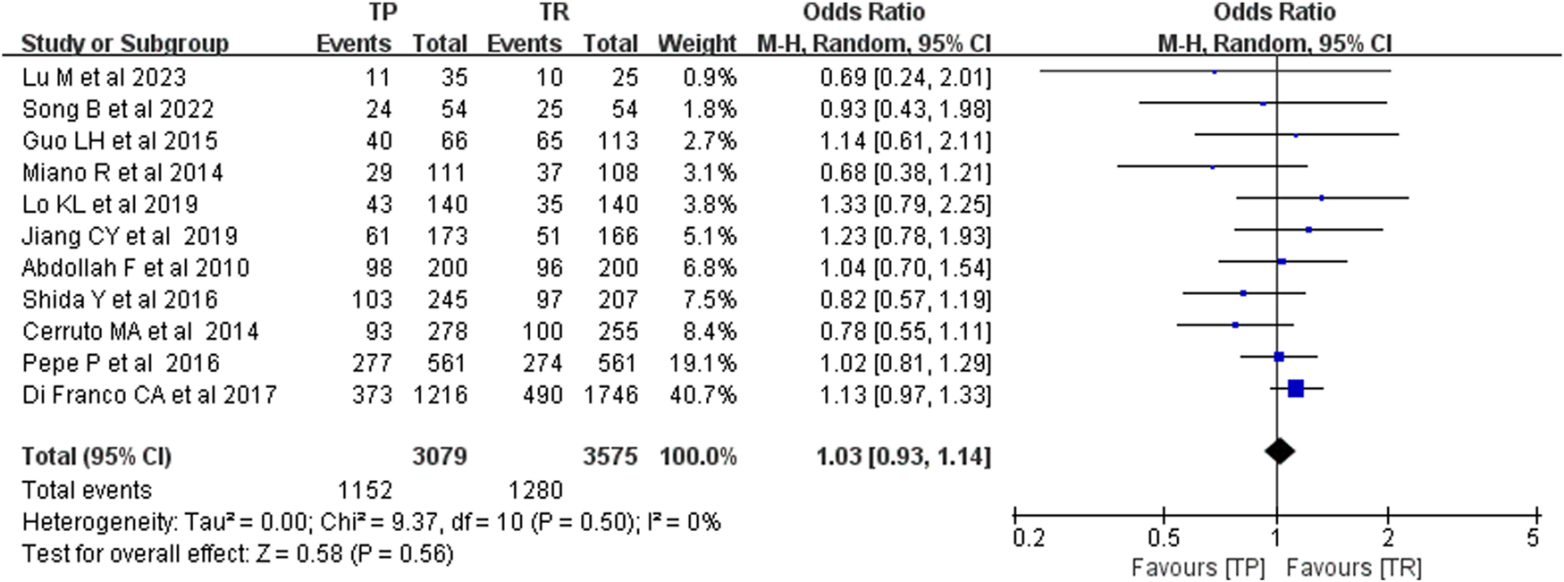
Prostate cancer diagnosis rate comparison.

Eleven eligible trials were identified that examined PCa diagnosis rates, and no publication bias was detected through funnel plot analysis. Analysis using the fixed-effect model revealed that the diagnosis rate for PCa by TP biopsy was comparable to that by TR biopsy, with no statistically significant difference (*P* < 0.005). The mean difference (MD) was 1.03, with a 95% CI of 0.91–1.14, and a *P*-value of 0.56. The heterogeneity of the literature had no significant impact on sensitivity. Furthermore, by examining the original literature, it was observed that there was no significant difference in sample size or experimental duration among these 11 studies. This finding is illustrated in Figure 2.

### 3.4 Prostate volume comparison

**Figure 3:**
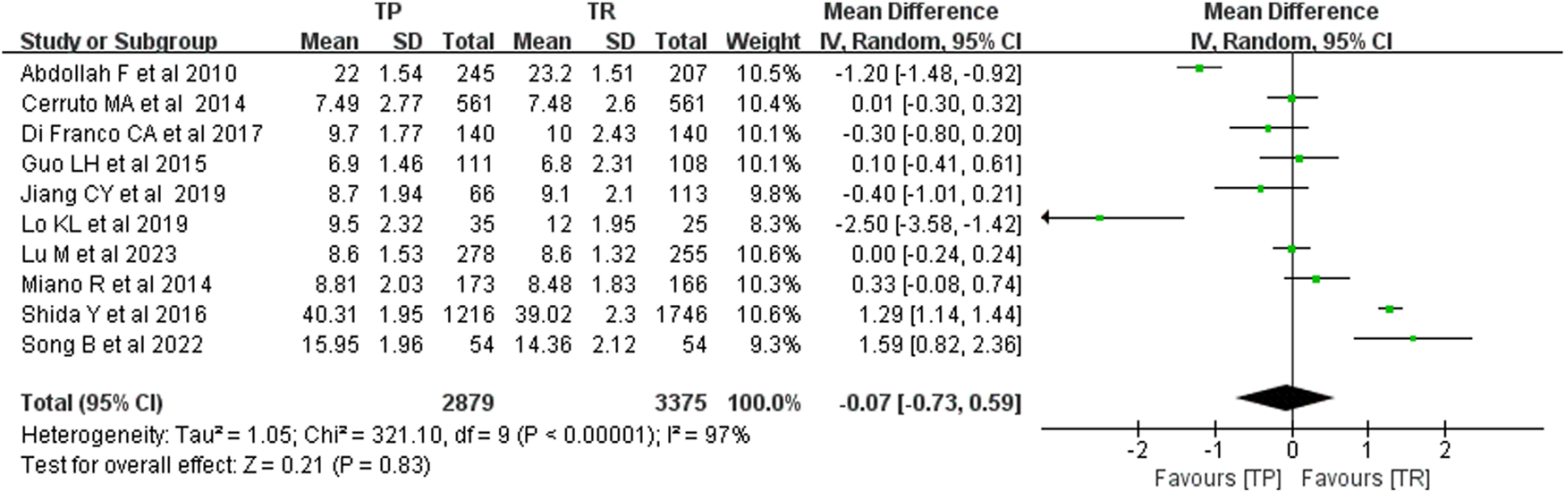
Prostate volume comparison.

We examined the prostate volume of patients diagnosed with PCa through ultrasound-guided TP and TR prostate biopsy. No significant publication bias was identified using funnel plot analysis. The comprehensive results from the random effects model indicated that the prostate volume in the TP group was similar to that in the TR group, with no statistically significant difference (*P* < 0.05). The MD was –0.07, with a 95% CI of –0.73 to 0.59, and a *P*-value of less than 0.0001. The forest plot revealed a combined effect size *P*-value of 0.83, suggesting no distinction in prostate volume between patients diagnosed with PCa via TP and TR biopsies. This is illustrated in Figure 3.

### 3.5 PSA level comparison

**Figure 4:**
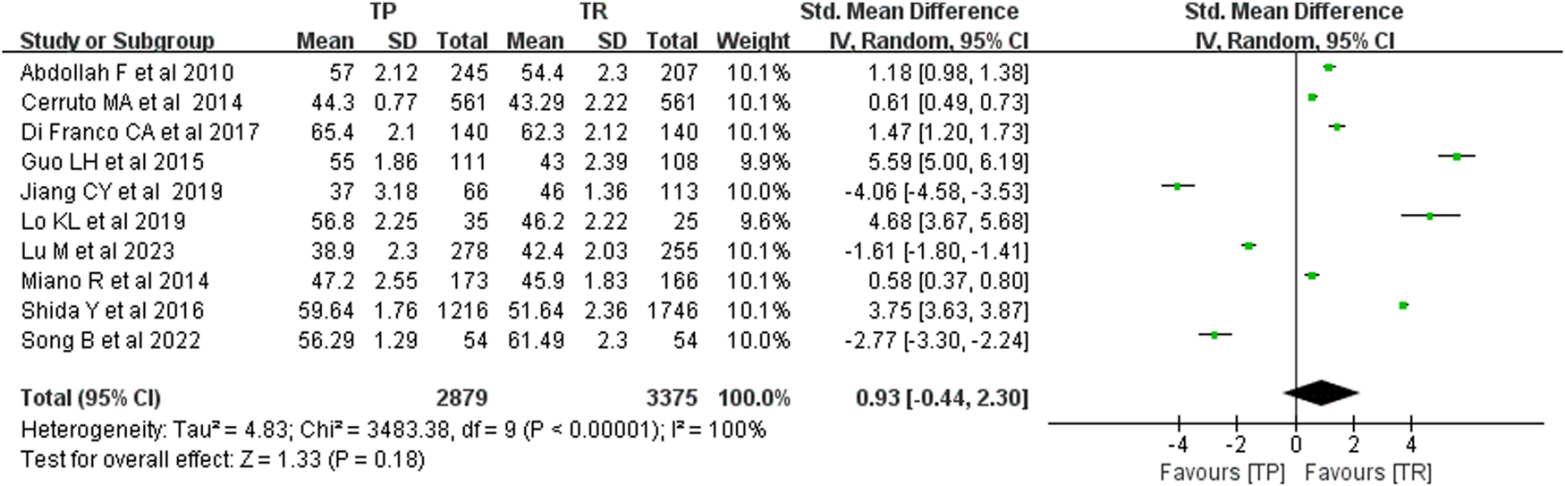
PSA level comparison.

We investigated PSA levels in patients diagnosed with PCa through ultrasound-guided TP and TR prostate biopsy. Analysis using the funnel plot method revealed no significant publication bias. The results obtained from the random effects model demonstrated that the PSA levels in the TP group were comparable to those in the TR group, with no statistically significant difference (*P* < 0.05). The MD was 0.93, with a 95% CI ranging from –0.44 to 2.30, and a *P*-value of less than 0.0001. The forest plot indicated a combined effect size *P*-value of 0.18, suggesting no variance in PSA levels between patients diagnosed with PCa via TP and TR biopsies (see Figure 4).

## 4 Discussion

The two primary techniques for prostate biopsy are TR and TP biopsy. The detection rates for PCa and overall complication rates are generally comparable for both methods, although it is worth noting that TR biopsy is more widely practiced globally. This is due to the fact that TR prostate biopsy offers the advantages of shorter procedure times, relatively simpler procedures, and the absence of the need for complex anesthesia. The American Urological Association and the European Association of Urology recommend TR biopsy as the most commonly used method, with TP biopsy serving as an effective alternative [25]. Research conducted by Udeh et al. revealed that TP biopsy did not demonstrate a significant advantage in detecting cancer during prostate biopsy, but it did exhibit a higher detection rate of core cancer in biopsy [13]. TP and TR biopsies showed similarities in PCa detection rates, suggesting that both methods have good results in diagnosing PCa. The diagnostic accuracy of TP biopsy is no less than TR biopsy, eliminating concerns about the effectiveness of TP biopsy. TP biopsy is especially suitable for patients who are concerned about the risk of infection associated with TR biopsy, as it can significantly reduce the risk of infection and rectal bleeding. Especially in a modern medical environment focused on patient safety and comfort, TP biopsy has emerged as a viable alternative to TR biopsy.

We included 11 trials that compared TP versus TR biopsy for the diagnosis of PCa and found no evidence of publication bias. When analyzed using a fixed-effect model, there was no significant difference in the detection rates between TP and TR biopsies (*P* < 0.005). The MD was 1.03, with a 95% CI of 0.91–1.14 and a *P*-value of 0.56. Our findings align with those of Xue, who concluded that there was no significant difference in the detection rate of PCa between TP and TR methods [8]. TR and TP biopsy techniques utilize different procedures, but both are capable of sampling prostate tissue for pathological testing to diagnose PCa. Whether employing TR or TP biopsies, the objective is to obtain a prostate tissue sample for pathological analysis to ascertain the presence, type, and extent of PCa. Therefore, in theory, the diagnostic efficacy of the two methods should be comparable. While there are procedural differences between TR and TP biopsies, both are performed under the guidance of a medical professional. Both procedures require attention to aseptic technique, patient comfort, and other relevant considerations to ensure the accuracy and reliability of the biopsy results.

To further elucidate the differences between the two biopsy methods, we conducted statistical analyses of serum PSA levels and prostate volume. The meta-analysis revealed that, when employing the random effects model, the prostate volume in both TP and TR biopsy groups was similar, with no statistically significant difference (*P* < 0.05). The MD was –0.07, with a 95% CI of –0.73 to 0.59, and a *P*-value of less than 0.0001. This indicates that there was no difference in prostate volume between patients diagnosed with PCa who underwent TP or TR biopsy. Additionally, we examined PSA levels in patients diagnosed with PCa via ultrasound-guided TP and TR biopsy and found no publication bias. The comprehensive results from the random effects model again showed that the prostate volume in the TP group was comparable to that in the TR group, with no statistically significant difference (*P* < 0.05). The MD was 0.93, with a 95% CI ranging from –0.44 to 2.30, and a *P*-value of less than 0.0001. The combined effect size *P*-value was 0.18, suggesting no variance in PSA levels between TP and TR biopsy patients diagnosed with PCa. Prostate volume is a critical factor that influences biopsy outcomes. The impact of prostate volume on different biopsy techniques has not been conclusively established.

The meta-analysis revealed statistically significant differences in prostate volume between the two groups. This suggests that prostate volume may indeed affect the selection of biopsy protocols. For larger prostates, a more comprehensive and precise biopsy protocol may be necessary to prevent missed or erroneous diagnoses. For smaller prostates, a simpler and more expedient biopsy option could be considered. We speculate that prostate volume may be associated with the positive rate of PCa detection across different biopsy protocols. Larger prostates may facilitate the detection of PCa with various biopsy techniques, while smaller prostates might necessitate more detailed biopsy protocols to achieve the same level of detection. These hypotheses require further corroboration through additional clinical studies. Our meta-analysis indicates that prostate volume may impact the choice of biopsy protocol. We speculate that prostate volume may be related to the positive rate of PCa detection with different biopsy protocols. These insights have significant implications for guiding clinicians in selecting the most appropriate biopsy techniques and warrant further clinical validation.

Our study enjoys a higher degree of confidence in assessing the diagnostic accuracy of both TP and TR prostate biopsy methods due to the thoroughness of our online database search, the acquisition of all potentially relevant publications, and the strict adherence to the PRISMA guidelines. To enhance the accuracy and reliability of our findings, we employed stringent inclusion criteria. During the screening process, we included only RCTs and high-quality observational studies with a low risk of bias, as indicated by NOS scores exceeding 6. A retrospective study showed that TR ultrasound-guided prostate biopsy (TRUS), the gold standard for performing prostate biopsies, has a higher incidence of post-biopsy sepsis compared to TP [26]. In this study, the incidence of sepsis after TR needle biopsy was 0.36%, compared to 0.48% for TP. Urinary tract infections occur in both methods but at a lower rate. The most common complication was acute urinary retention, which occurred in 28 cases after TP. Another study noted the detection rate and safety of TR and TP needle biopsies for PCa [27]. This retrospective analysis showed no statistically significant difference in PCa detection rates between the two groups. However, the total complication rate of the TP group was lower than that of the TR group, the interval from puncture to radical operation and the operative time of radical operation were shorter than those of the TR group, and the intraoperative blood loss and intraoperative prostate tissue adhesion rate were lower than those of the TR group. TP and TR biopsies were comparable in tumor detection rates, but TP biopsies appeared to have lower complication rates, especially in terms of reducing bleeding and acute urinary retention. However, the results of these studies are not entirely consistent, so the specific circumstances of the patient and the experience of the doctor should be considered when choosing a biopsy method.

We take into account several limitations when interpreting the results. Only three RCTs were included in this study, which may limit the broad applicability and accuracy of the study. This study was observational and could not fully address potential confounders, such as free PSA, benign prostatic hyperplasia, or other unreported factors that could have influenced the conclusions. There are language limitations and screening methods in this study, which may lead to the omission of some unpublished relevant studies, thus affecting the reliability of the results. Therefore, further large (RCTS are warranted.

## 5 Conclusion

In conclusion, our updated systematic review and meta-analysis of nine studies revealed no substantial difference in overall PCa detection rates between TP and TR ultrasound-guided prostate biopsy approaches. The findings do not support the notion of inferior diagnostic accuracy of TP biopsy when compared to standard TR biopsy. TP biopsy may thus be considered a suitable alternative, particularly for patients who are apprehensive about infection risks associated with TR biopsy. Further adequately powered randomized controlled trials are necessary to comprehensively assess the comparative effectiveness and potential benefits of the two biopsy techniques.
